# Mid-term results of a cementless hip stem in femoral revision: how much diaphyseal press-fit do we need?

**DOI:** 10.1007/s00402-023-05191-4

**Published:** 2024-01-13

**Authors:** Dirk Herold, Axel Kuttner, Lutz Dreyer, Christoph Eingartner

**Affiliations:** 1 Caritas Hospital, Bad Mergentheim, Germany; 2 Aesculap AG, Tuttlingen, Germany

**Keywords:** Modular cementless revision hip stem, Revision hip arthroplasty, Bicortical fixation length, Bone healing

## Abstract

**Introduction:**

The aim of this retrospective study was to analyze the clinical and functional outcome of a modular tapered revision hip stem after mid-term follow-up with a special focus on the length of the distal bicortical fixation of the cementless hip stem.

**Materials and Methods:**

Follow-up examination was carried out for all patients with implantation of the Prevision hip stem between 2014 and 2019 to collect demographic, functional, and radiographic data.

**Results:**

44 patients with stem in situ were examined, and 61 patients could be included in the Kaplan–Meier survival analysis. Oxford’s hip score was 37.3 at the mean follow-up of 4.0 years. Two hip stem revisions were performed due to periprosthetic infection, which resulted in a hip stem survival rate of 96.7% (CI: 87.4–99.1%) at the final follow-up of 7.5 years. No aseptic hip stem revision was required. The length of bicortical distal fixation was in the interquartile range of 6.8 to 9.0 cm, which was associated with good bone healing and a low rate of subsidence (4.5%). Implant-associated complications were observed in 10 cases (21.7%).

**Conclusions:**

The modular revision hip stem provides promising results at medium-term follow-up, with satisfactory clinical and functional outcomes comparable to other modular revision hip stems. The presented length of bicortical distal fixation shows the practice of the study center and was associated with good implant survival, bone healing and radiological results.

**Registration:**

Clinicaltrials.gov registration: NCT04833634 registered on April 6, 2021

## Introduction

Compared to standard primary total hip arthroplasty (THA), the revision of a hip joint prosthesis is a more technically complex and clinically challenging procedure. In the presence of moderate to severe bone defects, a modular or monobloc revision stem with cementless distal fixation is often indicated. Modular revision stems are two-part hip stems with a modular connection, and therefore allow independent distal and proximal size selection, and also allow free rotational assembly for more flexibility in stem anteversion. Modularity always is linked to higher complexity, therefore the surgical technique is more demanding than monobloc revision stems, but may offer more length and diameter options for a secure distal fixation [1]. In the second step, the selection of the proximal component allows an adaptation to the proximal bone situation for maximal proximal support, with the possibility for adjustment of offset and anteversion [2, 3]. Despite these technical features, the superiority of modular over monobloc hip revision stems has not yet been convincingly proven: In a systematic review of studies of monobloc and modular tapered fluted hip stems with over 4,000 stem revisions, similar re-revision rates, dislocation rates, periprosthetic fracture rates and infection rates were observed in both groups [4]. Significant differences were only detected in rates of subsidence (in favor of modular stems) and rates of periprosthetic fracture (where monobloc stems showed better results). Similar results were also observed from a more recent systematic review including a total of 2188 hips [5].

Therefore, the choice of a suitable revision implant is still based on surgeon preference and treatment goals. Modular implants allow a more precise adjustment of the distal stem length and hence also the desired length of distal fixation [6]. The primary stability of the distal fixation is especially important with the transfemoral technique, where an osteotomy is prepared for removal of the previous stem. However, scientifically based guidelines for the minimal length of required distal contact are lacking. The primary aim of the current retrospective study was to analyze the implant survival and functional outcome of a cementless modular tapered revision hip stem at mid-term follow-up. Particular attention was paid to the length of distal fixation with bicortical stem contact, and its possible impact on the functional outcome and implant subsidence.

## Materials and methods

### Study population and retrospective data collection

Follow-up examination was carried out in 2021 and 2022 for all patients operated on with the Prevision hip stem between 2014 and 2019. Analyses included demographic data and indication for the hip stem implantation, complications, and mobility-restricting factors at follow-up.

This study was performed in line with the principles of the Declaration of Helsinki, and all patients provided written informed consent. Approval was granted by the competent ethics committee (Landesärztekammer Baden-Württemberg, F-2020–170).

### Surgical procedure

All procedures were conducted by one of four senior surgeons. Before the surgery, a joint aspiration was performed for leukocyte count and synovial fluid culture, and intraoperative samples were taken for microbiological and histological analyses. In case of suspected periprosthetic joint infection (PJI), the removed foreign material was sent to sonication.

Revisions due to PJI generally were treated with a two-stage replacement and a spacer interval of 6 to 8 weeks. Antibiotic therapy was carried out following the Pro-Implant treatment algorithm [7]. In aseptic revisions, 2 g of basic cephalosporin (Cefazolin) was routinely administered intravenously as antibiotic prophylaxis, repeated 2 h after surgery. A cell-saver system was used only in the absence of PJI.

Procedures were planned preoperatively (mediCAD Hectec, Germany) to determine the length and location of the osteotomy as well as the required implant components, with the aim of at least 8 cm of distal fixation. The Prevision modular revision prosthesis (Aesculap, Germany) is available with nine different proximal components. Distal components have a star profile with longitudinal flutes for rotational stability and are available in diameters from 12 to 24 mm and in lengths from 200 to 400 mm. Straight distal components have a 2° conical shape, and curved stem have a subproximal 4° conical shape and a distal cylindrical shape.

Surgery was performed by the transfemoral technique via a direct lateral approach in supine position. Osteotomy was performed similarly to the extended trochanteric osteotomy (ETO) [8]. While in ETO the bony flap takes 1/3 of the stem circumference, in the here used transfemoral approach the bony flap is more extensive and its width covers half the circumference of the stem as described by Wagner [9, 10]. The transfemoral approach was also chosen because of the local need to revise a large proportion of an extensively porous-coated hip stem, which is known to be very difficult to remove even when loose [10]. It also offers the possibility to adapt the proximal femur to the implant, so that in all but one case a straight distal component could be used.

According to the preoperative planning, the osteotomy was usually placed at the tip of the previous hip stem, or at the end of the coated surface. After the application of a distally placed safety cerclage with double wire, the osteotomy was started with several drill holes and completed with an oscillating saw and chisels. The distal fixation bed was prepared with conical reamers of rising diameter until a good cortical press fit and the required depth of insertion was reached. Routinely, the last reamer was controlled under fluoroscopy regarding the length of distal fixation, endostal pressfit and absence of periprosthetic fracture. The length, diameter and anteversion of the proximal component and head length were determined with trial implants. The final proximal component was connected in situ, and the osteotomy was closed with wire cerclages. If necessary, the greater trochanter fragment was reattached by wiring to the proximal component. The follow-up treatment included usually full weight bearing with a walker or crutches and limitation for hip flexion over 90°.

### Functional and radiological outcome

Functional outcome at follow-up was measured by Oxford Hip Score (OHS), with higher values for better outcomes (current scoring system). If necessary, OHS values reported from the literature were converted accordingly in the discussion section. Preoperative values were not available. In addition, patient satisfaction was asked on a 4-part scale from “very dissatisfied” to “very satisfied”.

The length of distal stem fixation, which was defined as the length of distal bicortical contact of the hip stem, was measured from postoperative anterior–posterior and lateral X-rays as reported elsewhere [2]. Stem subsidence at follow-up was measured in relation to the direct postoperative X-rays.

Radiologic analysis at follow-up included assessment of the following criteria: qualitative assessment of distal radiographic bone ingrowth and proximal remodeling, presence of radiolucent lines > 2 mm around the stem, bone resorption or hypertrophy in the distal fixation zone and (if applicable) assessment of bone union of the osteotomy and healing of the fracture. If any abnormality was found, these patients were rated as “poor radiological bone remodeling”.

### Statistical analysis

All patients with signed informed consent and all available information for the deceased patients were included for Kaplan–Meier survival, with the endpoints “stem survival” (primary endpoint) or “all component survival”. 95% confidence intervals (CI) were calculated for the Kaplan–Meier estimates of the survival rate.

A correlation between distal fixation length and stem subsidence was analyzed by Pearson correlation coefficients. The relations between functional outcome and indication were investigated using multiple mixed linear regression; age, gender and BMI were used as additional patient-based predictors of the regression model. A difference in OHS outcome between normal and poor radiological bone remodeling patients was analyzed by t-test for independent groups. Methods for analyzing normally distributed data (Pearson correlation, t-test) were used when data distribution did not contradict the normality assumption in scatter plots. All analyses other than survival analysis only included patients with hip stem in situ at follow-up. Standard deviation (SD) was calculated as a variability measure for quantitative values.

## Results

### Study population and patient characteristics

**Fig. 1:**
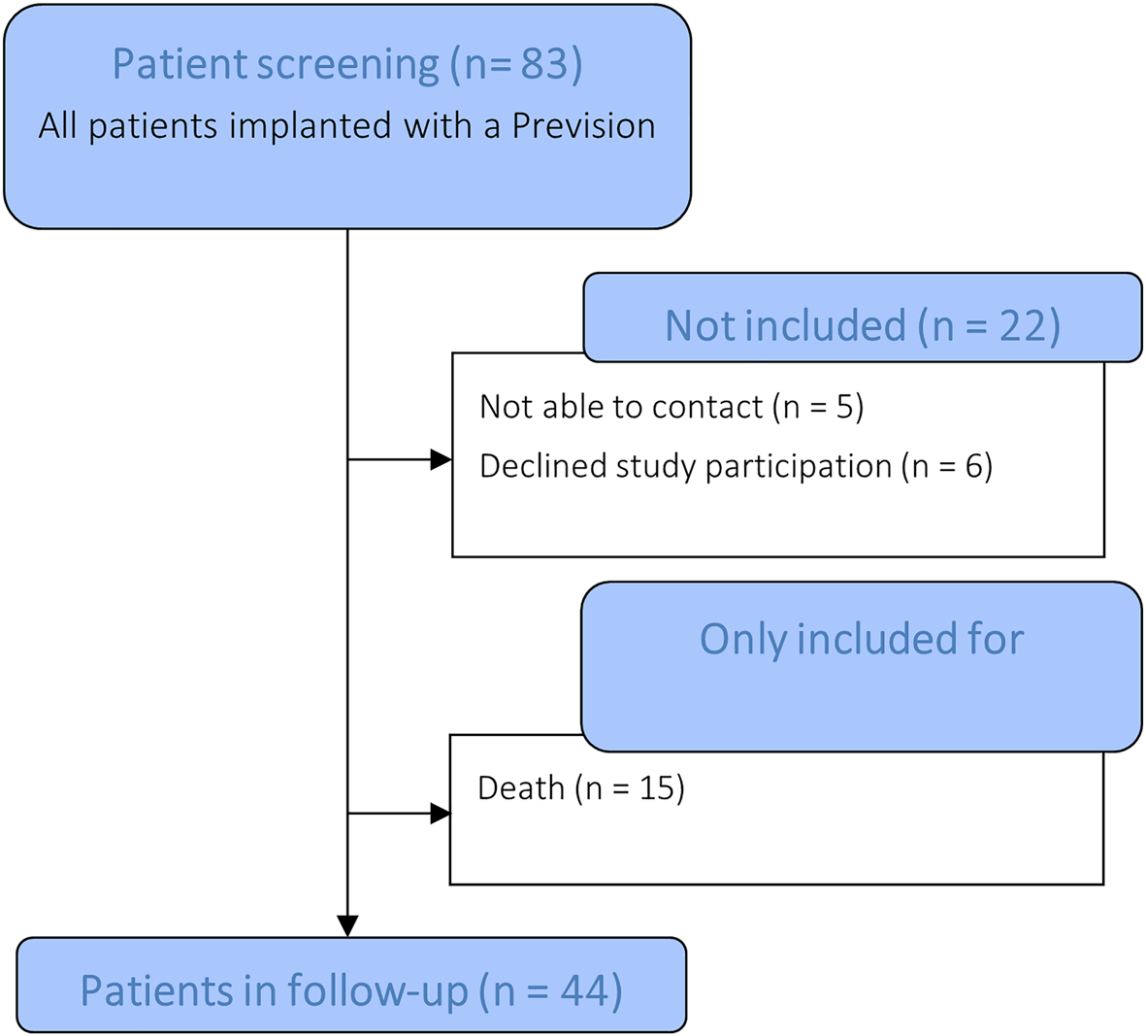
Patient inclusion flowchart. The Kaplan–Meier analysis of stem survival was based on the patients in follow-up together with the stem-revised and deceased patients (*n* = 61)

In total, all 83 patients with implantation of the Prevision hip stem at the study center between 2014 and 2019 were screened for study participation (see Fig. 1). 44 patients with stem in situ could be included. The clinical follow-up of the study patients was performed during the COVID-19 pandemic, therefore in some cases, where current x-rays from the study site or from resident doctors were available, a telephone interview was accepted. A physical examination was performed in 35 of the 44 patients, who were included at the follow-up. Mean follow-up was 4.0 years (SD 1.6 years).

In total, 22 patients could not be contacted, declined study participation or were not able to participate in the follow-up examinations. 15 of the screened patients died since the surgery. Of these, two cases occurred during the postoperative inpatient stay which were not implant-associated. From the deceased patients, and from patients not included in the study, no hip stem revisions were reported or otherwise known to the investigators. For Kaplan–Meier survival analysis, the latest known implant status of the deceased patients was used based on information either from patient records, from relatives or the treating general practitioner.

Mean age of the patients at surgery was 70.1 years, with a mean BMI of 27.6 kg/m^2^. Indications for stem implantation were mostly THA revision (see Table 1), which was performed due to PJI (45.5%), aseptic loosening (29.5%) or periprosthetic fracture (18.2%).

**Table 1:** Indication for stem implantation

Indication	*n* (%)
Periprosthetic joint infection	20 (45.5%)
Aseptic loosening	13 (29.5%)
Periprosthetic fracture	8 (18.2%)
Failed osteosynthesis	2 (4.5%)
Intraoperative periprosthetic fracture	1 (2.3%)
Sum	44 (100%)

The procedure was mostly the first revision (85.3% of replaced hip stems) or in other cases the second revision. The previous hip stem was cementless in 90% of the cases. Mean duration of the surgery was 165.7 min (SD 45.8 min), and the length of hospital stay was 14.7 days (SD 5.0 days).

Straight distal components were implanted in all but one case of a curved stem. Median distal component diameter was 14 mm (range 12–20 mm), median distal component length was 240 mm (range 200–320 mm). In most of the cases (79.5%), a ceramic head was used.

Distal locking screws were used in only one patient, in a THA revision after aseptic loosening.

### Risk factors and comorbidities

Risk factors and mobility-restricting factors at follow-up were recorded for the patients. For 93.2% of the patients, at least one factor was documented. 52.2% of the patients had implants or other impairments at the contralateral hip. Further mobility-restricting factors concerned other joints (40.9%), the spine (38.6%), cardiovascular factors (36.3%) and others (34.0%).

### Complications

A total of 17 complications were recorded (see Table 2), which affected ten patients. Ten of the complications were implant-associated; one intraoperative fracture occurred at the removal of the cement spacer and before implantation of the hip stem, and was therefore not rated as implant-associated complication.

**Table 2:** List of complications

Implant-associated complications:	
Dislocation	5
Secondary trochanter dislocation	2
Infection	2
Aseptic loosening (acetabular cup)	1
General surgery complications:	
Hematoma	3
Neurological complication	3
Intraoperative fracture	1

Twelve of the complications led to reoperation, and in seven of these, a partly or complete exchange of implant components was performed. Reason for revision included dislocation (*n* = 3), PJI (*n* = 2), trochanter dislocation (*n* = 1), and aseptic loosening of the cup (*n* = 1). Cup loosening was observed in a patient who had already experienced a revision of the cup due to dislocation, meaning the seven implant revisions affected six patients.

### Survival analysis

**Fig. 2:**
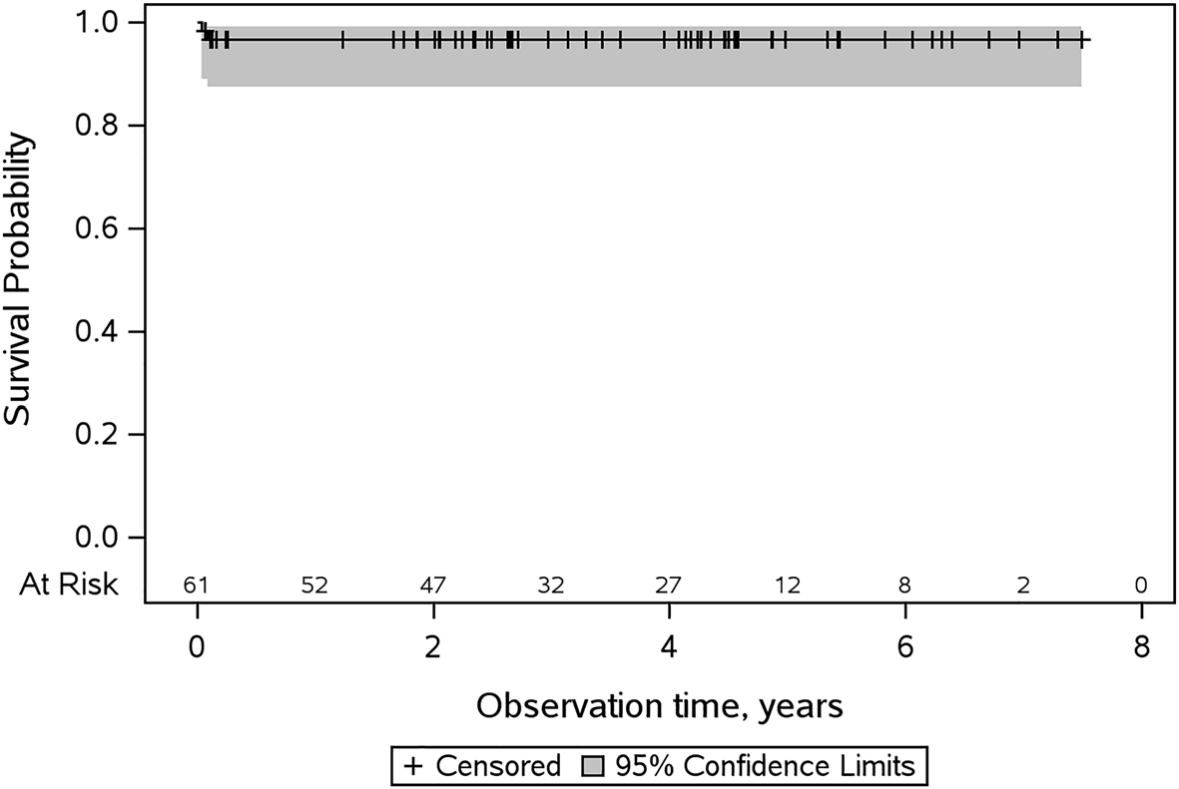
Kaplan–Meier survival of the hip stem, with 95% confidence intervals and 96.7% (CI: 87.4–99.1%) survival rate after 7.5 years. The number of patients at risk is shown on the bottom line

A hip stem revision was performed in two cases, which were both due to early PJI, one recurrent, and required complete component removal, which were made at 15 and 33 days after implantation, respectively. Implant survival with the endpoint stem revision was therefore 96.7% (CI: 87.4–99.1%) at the mean follow-up of 4.0 years, with no changes until the maximum follow-up of 7.5 years (see Fig. 2).

**Fig. 3:**
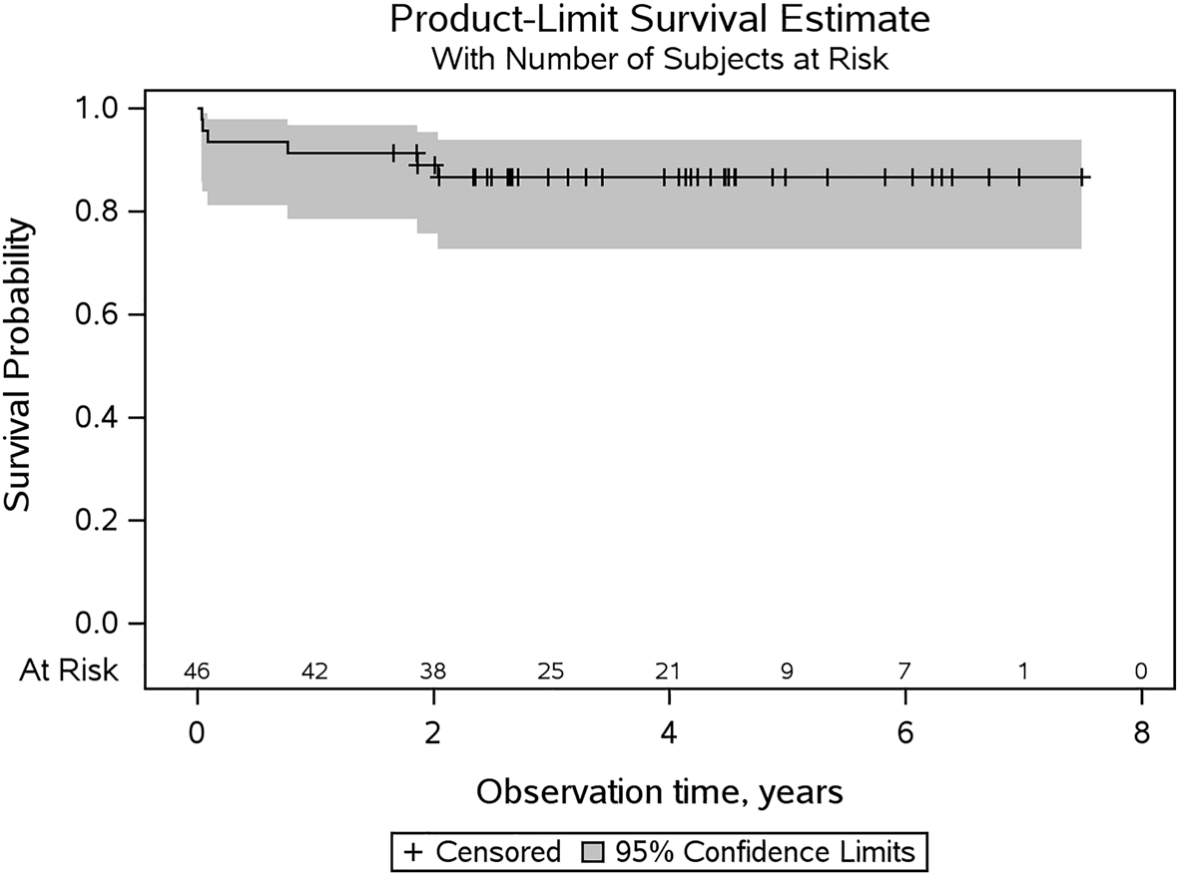
Kaplan–Meier survival with the endpoint “any implant component revision”, with 95% confidence intervals and 86.6% (CI: 72.5–93.7%) survival rate after 7.5 years. The number of patients at risk is shown on the bottom line

In addition to these complete component exchanges, isolated exchange of the prosthesis head was performed in three cases, as well as two isolated cup revisions in one patient (accounting for one event in Kaplan–Meier analysis), which results in a survival rate (any implant component) of 86.6% (CI: 72.5–93.7%), also both at the 4.0 and at the 7.5-year follow-up (see Fig. 3).

### Functional and radiological outcome

At follow-up, 43 of 44 patients (97.7%) stated that they were “very satisfied” or “satisfied” with the result of the hip surgery. Mean OHS was 37.3 (SD 10.2), with no significant correlation between the different indications for stem implantation.

A limp (Duchenne or Trendelenburg gait) was diagnosed in 16 of 38 patients. At follow-up, a leg length difference of the treated leg was observed in 21 patients (47.7%), ranging from − 1.5 to + 2.0 cm, with no tendency to a systematic lengthening or shortening (mean difference in the 21 patients was − 0.1 cm).

**Fig. 4:**
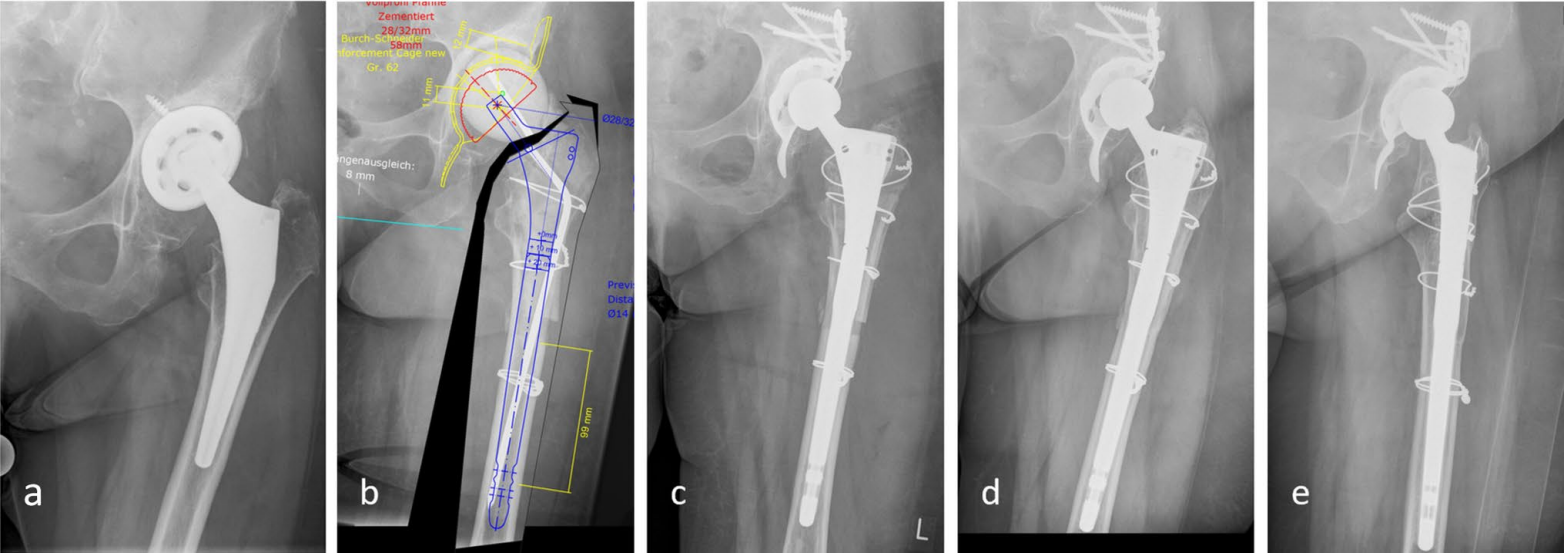
Two-stage hip revision of a 62-year-old female patient with septic loosening of the cup and a well-fixed cementless hip stem. a) Condition before revision. b) Preoperative planning with length of distal fixation with spacer implanted. c) Postoperatively with cemented cup and Burch Schneider reinforcement cage. d, e) Anteroposterior and axial radiographs 4 years postoperatively, with healed osteotomy and good proximal remodelling

Figure 4 shows a case of a 62-year-old female patient with periprosthetic joint infection, treated with a two-stage exchange with a femoral stem revision as described above.

**Fig. 5:**
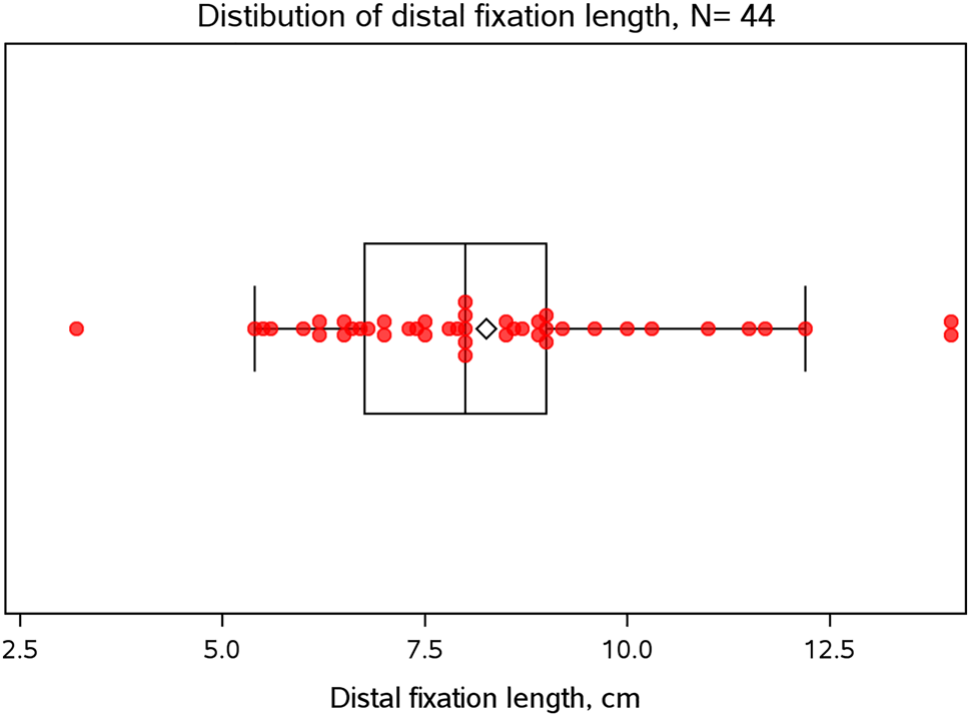
Length of distal fixation, determined from directly postoperative X-rays. Mean fixation length was 8.3 cm (SD 2.2 cm)

The mean length of distal fixation in the initial postoperative X-rays was 8.3 cm (SD 2.2 cm), ranging from 3.2 to 14.0 cm (see Fig. 5). The interquartile range, which describes the spread of the middle 50% of the determined values, was between 6.8 and 9.0 cm.

Fracture healing and consolidation of the osteotomy were observed in all cases, with no radiolucent lines present in the patients. Four patients were rated with “poor radiological bone remodeling”, which was in all cases due to poor proximal remodeling and in three cases additionally due to a non-union of the *Trochanter major*. Functional outcome in patients with poor radiological bone remodeling was non-significantly lower than in the other patients, with an OHS of 32.0 vs. 37.8, respectively.

In two patients (4.5%), a stem subsidence was observed, which was 4 mm and 6 mm respectively. No other radiologic abnormalities were found in these patients, and OHS score was 25 and 37. The length of distal fixation was regular (8.5 cm and 8.7 cm respectively). Thus, no correlation between the length of distal fixation and subsidence could be found.

## Discussion

This study describes the clinical and radiological outcome of a cementless modular revision hip stem in revision THA at a mid-term follow-up of 4.0 years. The patients had multiple preexisting comorbidities and risk factors, with the presence of at least one mobility-restricting factor in over 90% of the patients. Despite this complex setting, the patient satisfaction with the surgical results was generally high at 97.7%. The OHS at follow-up was 37.3, which was comparable to a reported OHS of 32 after fractures reported by Scalici et al. [11], of 34 at two-year follow-up reported by Willems et al. [12], of 32.07 at six years follow-up by Abdelsamie et al. [1], or 34 after five years by Lequeuche et al. [13] in revision THA.

Ten implant-associated complications (21.7%) were observed, which is comparable to results for other revision hip stems by Willems et al. with a complication rate of 13% [12], Valtanen et al. of 24.7% [14], or Park et al. of 44.4% [15]. The most frequent complication was dislocation, which led to reoperation in three patients (6.8%) with the exchange of the acetabular cup or isolated exchange of the prosthesis head.

In the patient cohort for this retrospective study, a dual mobility system (Avantage, Zimmer-Biomet, USA) was only used in 3 cases. As a result of the cases presented here as well as based on favorable results in the literature for revision THA [16, 17], dual mobility articulation has been used increasingly in our center in recent years. A similar tendency can also be observed in the German arthroplasty registry which led to a rise in dual mobility articulations in revision THA from 10% in 2014 to over 30% in 2021 [18, 19].

In our series, hip stem survival was 96.7% (CI: 87.4–99.1%) at 7.5 years, which is very satisfactory. All-component survival was 86.6% (CI: 72.5–93.7%). In both cases of stem revision, the underlying cause was an infection, one of these recurrent. No aseptic revision had to be performed in our series.

At radiologic follow-up, all fractures and osteotomies completely healed. This is in line with previously reported good results in femoral revision in Vancouver B2 and B3 fractures [15]. In our series, proximal bone remodeling was rated “poor” in four patients, but there was no correlation to inferior clinical outcomes. Leg length discrepancies were acceptable, ranging from − 1.5 to + 2.0 cm, which might be a positive effect of the modularity.

No correlation between the length of fixation and the two cases (4.5%) of stem subsidence, or any impact on the clinical outcome were observed. The length of bicortical distal fixation was typically in the range of 6.8 to 9.0 cm (interquartile range). This is less than the recommendation of at least 10 cm by the manufacturer, but also considerably longer than the previously reported 4.11 ± 0.92 cm [2]. In this study, satisfactory results were reported from an analysis of 140 modular revision stems with a distal 2° taper, similar to the hip stem used in this study. A significant subsidence was observed in only 2.1% of patients after transfemoral implantation, and no aseptic revision was required at a mean follow-up of 7.4 years [2]. In a similar study, 5.5 cm of bicortical contact was reported both for monobloc and modular cementless hip stems, with similar good rates of significant subsidence of 2.7% and 3.7%, respectively [20].

In a retrospective comparison of two modular tapered revision stem designs, Pawar et al. presented low complication rates and good survival after 5 years [21]. However, comparably high rates of subsidence were observed with the two stem designs: a > 5 mm subsidence was detected in 10% of patients treated with the Arcos stem, and 30% of the patients treated with the Reclaim stem. As no influencing factor other than stem design was found in the study, the authors hypothesized that the higher rates of subsidence could be related to the low taper angle of 2.5° of the Reclaim stem. This hypothesis was also based on a discussion of results from the literature. The findings however are not in line with the current study, where a modular stem with a 2° distal taper angle showed subsidence in only 4.5% of the patients after a comparable follow-up.

To conclude, the comparably high length of distal fixation in the study population can be considered safe for primary stem fixation. The research question however about the necessary minimal length of bicortical contact for this hip stem design cannot be answered based on these data and only two cases of subsidence, which were not connected to a small length of distal fixation. The combination of modularity and a transfemoral approach allows sound distal fixation under direct visual and tactile control, without compromises with respect to the articulation: length, anteversion and offset can be adjusted independently at a later stage of the operation. Thus, good primary stability and diaphyseal press-fit might be important factors for low subsidence rates.

Distal interlocking was not performed in our series. It may be considered if a deficiency of the isthmus is present, classified as Paprosky IIIB or IV, or Vancouver B3 in fracture cases. When using interlocking screws, the difference in angularity of the straight distal component with 2° taper and of the curved distal component with distal cylindrical shape must be noted. With a lower taper, the fixation relies heavily on the locking screws, possibly leading to a higher rate of screw breakage, subsidence and loosening [22].

The current study has some limitations. First, the study is retrospective, and only slightly more than half of the eligible patient cohort was able to participate in the follow-up examination. In addition, the mortality was high with 18% at follow-up. This may be explained by the fact that 19 of the 83 patients eligible for this retrospective study were 80 years of age or older at the time of surgery. In addition, most patients (68.2%) had non-elective surgery. Of the patients who refused participation or could not be contacted, no case of additional stem revision was known from the patient screening, which, however, cannot be ruled out. Therefore, these patients were not included in the Kaplan–Meier estimation. Second major limitation is that a functional score was not routinely obtained before implantation. This would help to interpret the OHS values at follow-up, which are clearly inferior to primary hip arthroplasty results. And lastly, the analysis of the length of distal fixation was based on lateral and anteroposterior radiographs. Postoperative CT scans would have facilitated the measurements but were not available for the study patients.

In conclusion, the clinical and radiological outcomes of femoral revision with a straight modular stem are encouraging and comparable to other modular revision hip stems. Due to the lack of conspicuous patients with regard to radiological or clinical results, the question of a recommendable minimum diaphyseal anchorage distance still remains unanswered based on the presented data. Further clinical and biomechanical research is encouraged to define a minimum safe length of distal fixation of distal fixating revision hip stems. The length of bicortical distal fixation between 6.8 and 9 cm was associated with good implant survival and low rates of subsidence, without the need for distal interlocking.

## Data Availability

Data is available upon reasonable request.
